# A link between circulating immune complexes and acute kidney injury in human visceral leishmaniasis

**DOI:** 10.1038/s41598-024-60209-0

**Published:** 2024-04-30

**Authors:** Gabriela Corrêa-Castro, Maria Luciana Silva-Freitas, Ludmila de Paula, Leonardo Soares Pereira, Maria Rita Teixeira Dutra, Hermano Gomes Albuquerque, Glaucia Cota, Caroline de Azevedo Martins, Alda Maria Da-Cruz, Adriano Gomes-Silva, Joanna Reis Santos-Oliveira

**Affiliations:** 1 Laboratório Interdisciplinar de Pesquisas Médicas, Instituto Oswaldo Cruz, FIOCRUZ, Rio de Janeiro, Brazil; 2 Núcleo de Ciências Biomédicas Aplicadas, Instituto Federal de Educação, Ciência e Tecnologia, IFRJ, Rio de Janeiro, Brazil; 3 Hospital Eduardo de Menezes, Fundação Hospitalar do Estado de Minas Gerais, Minas Gerais, Brazil; 4 Laboratório de Doenças Parasitárias, Instituto Oswaldo Cruz, FIOCRUZ, Rio de Janeiro, Brazil; 5 Instituto René Rachou, FIOCRUZ, Minas Gerais, Brazil; 6 Disciplina de Nefrologia, Faculdade de Ciências Médicas, UNIRIO, Rio de Janeiro, Brazil; 7 Disciplina de Parasitologia, DMIP, Faculdade de Ciências Médicas, UERJ, Rio de Janeiro, Brazil; 8 Rede de Pesquisas em Saúde do Estado do Rio de Janeiro, FAPERJ, Rio de Janeiro, Brazil; 9 Instituto Nacional de Neuroimunomodulação, INCT–NIM-CNPq, Rio de Janeiro, Brazil; 10 Laboratório de Pesquisa Clínica em Micobacterioses, Instituto Nacional de Infectologia Evandro Chagas, FIOCRUZ, Rio de Janeiro, Brazil

**Keywords:** Parasitic infection, Antibodies

## Abstract

Visceral leishmaniasis (VL) is an infectious disease caused by *Leishmania infantum*. Clinically, VL evolves with systemic impairment, immunosuppression and hyperactivation with hypergammaglobulinemia. Although renal involvement has been recognized, a dearth of understanding about the underlying mechanisms driving acute kidney injury (AKI) in VL remains. We aimed to evaluate the involvement of immunoglobulins (Igs) and immune complexes (CIC) in the occurrence of AKI in VL patients. Fourteen VL patients were evaluated between early treatment and 12 months post-treatment (mpt). Anti-*Leishmania* Igs, CIC, cystatin C, C3a and C5a were assessed and correlated with AKI markers. Interestingly, high levels of CIC were observed in VL patients up to 6 mpt. Concomitantly, twelve patients met the criteria for AKI, while high levels of cystatin C were observed up to 6 mpt. Plasmatic cystatin C was positively correlated with CIC and Igs. Moreover, C5a was correlated with cystatin C, CIC and Igs. We did not identify any correlation between amphotericin B use and kidney function markers in VL patients, although this association needs to be further explored in subsequent studies. Our data reinforce the presence of an important renal function impairment during VL, suggesting the involvement of Igs, CIC, and C5a in this clinical condition.

## Introduction

Visceral leishmaniasis (VL), also known as kala-azar, is a systemic and severe infectious disease. In Brazil, VL is caused by the *Leishmania (L.) infantum* parasite, which is transmitted to mammals through the bites of *Lutzomyia longipalpis* sand flies. Moreover, VL is reported in 83 countries worldwide, with 30,000 new cases occurring annually^1^. Brazil is one of the countries with the most cases of VL, accounting for 93.5% of 1799 cases reported in the Americas in 2021^2^. Furthermore, the highest VL case fatality was reported in Brazil, with an average of 8.4% between 2007 and 2017^3^. Belo Horizonte (Minas Gerais state) is among the five Brazilian municipalities with the greatest number of VL cases^2^, with 2472 cases reported between 2007 and 2020^4^.

VL manifests across a spectrum of clinical presentations, varying from asymptomatic to severe and fatal disease, particularly when left untreated^5^. Symptoms such as recurrent fever, hepatosplenomegaly, asthenia, anorexia, and weight loss are frequently reported during active disease^6,7^. Moreover, signs of impaired kidney and liver functions have been described^8^ and may be linked with death in VL^5^. Patients typically present with severe anemia and pancytopenia, associated with high levels of C-reactive protein and gamma globulins^5,9, 10^. In addition, active VL is characterized by immunopathogenic disorders, notably the depletion of CD4^+^ T lymphocytes^11–13^, accompanied by an excessive inflammatory condition characterized by a cytokine storm and the polyclonal activation of T and B cells^7,12, 14, 15^.

Polyclonal activation of B cells in active VL is characterized by elevated levels of immunoglobulins (Igs), both specific or non-specific for the parasite^14,16, 17^. Nonetheless, the role of antibodies in the immune response against the parasite or in the pathogenesis of VL remains to be elucidated. Earlier studies demonstrated that antibodies are unable to prevent disease recurrence^18^ but may exacerbate the clinical evolution of VL^19–22^.

In this scenario, high circulating immune complexes (CIC) levels have also been described in human and canine VL cases^14,23–32^. Moreover, parasitic antigens have been observed in the composition of the CIC, and their evaluation was highlighted as an important diagnostic for VL^28,33, 34^. The formation of CIC is an inherent process of the humoral immune response, typically followed by several clearance mechanisms, such as complement system activation, facilitated transport by erythrocytes, and phagocytosis by macrophage in the liver or spleen^35^. However, excessive amounts of CIC may deposit in glomerular capillaries, skin, and vascular walls, causing severe injury^36,37^. This is more likely to occur in prolonged CIC formation or conditions with impaired clearance capacity^35^. In VL, several factors, including elevated levels of Igs, erythrocyte depletion, modulation of complement receptor 1 expression^38^, and impairment in phagocyte functions may contribute to the deposition of CIC.

In light of this, the association between elevated levels of CIC and renal damage has been widely explored in animal models. Notably, glomerulonephritis (GN) lesions, with IgG and IgM deposits were observed in 34 dogs naturally infected with *L. infantum*^39^. A case of canine VL with GN and interstitial nephritis associated with the presence of *L. donovani* in renal tissue has been reported. Interestingly, the histopathological characteristics resembled those of lupus-nephritis triggered by the deposition of immune complexes^40^. Several other studies have demonstrated renal damage in experimental VL, such as GN and tubulointerstitial injury in canine VL^41–45^ and *L. donovani* antigens along with Igs in glomerular lesions in infected hamsters^46^. Furthermore, time-dependent IgG deposits in kidney tissue were observed in infected mice^47^.

In humans with VL, several clinical signs of renal involvement have been described. In addition to acute kidney injury (AKI), urinary abnormalities, such as oliguria, proteinuria, hematuria, pyuria^6,48–50^, tubulointerstitial nephritis, and GN were described in patients with VL^49–56^. A prospective study demonstrated that approximately 60% of hospitalized patients with VL presented evidence of mild renal impairment. Interestingly, the histological evaluation in seven patients also demonstrated the occurrence of GN and interstitial nephritis^48^. Similarly, in a cohort of patients with AKI admitted to an intensive care unit in an endemic area in Brazil, 19.2% of patients were diagnosed with VL^57^, suggesting that in some cases, the renal injury draws attention even before the definitive diagnosis of VL^57,58^. Concurrently, 45.9% of AKI was reported in a cohort of children with VL, with almost 60% of them presenting renal dysfunction at admission, indicating a relation to the disease process itself^8^. Moreover, AKI at admission in adult cohorts with VL has been observed in other studies^50,54, 55, 59^.

Consistently with previous findings, increased cystatin C levels, but not creatinine, were observed in patients with VL^60^. Cystatin C, a protein belonging to cysteine protease inhibitors, is almost constantly produced by nucleated cells and is catabolized in the proximal renal tubule without reabsorption or secretion. Cystatin C has been considered a better marker of renal function due to its heightened levels in the early stages of AKI than other relevant markers^61–63^. Other studies reported a decrease in glomerular filtration rate (GFR) in patients with chronic VL, a phenomenon attributed to factors such as fluid loss, hypotension, and immunological glomerular disease^64^. Furthermore, proteinuria described in patients with VL predominantly consisted of low molecular weight protein fractions that migrated alongside gamma globulins^56^.

Notably, the specific VL treatment with amphotericin B should be monitored due to the occurrence of adverse events, particularly nephrotoxicity^55,59, 65–67^. Although with low rates of toxicity^68–72^, lipid formulations also warrant careful monitoring^73^. Previous studies have revealed an increase in serum creatinine levels and renal dysfunction in patients with VL under treatment^74^. However, histological alterations consistent with acute interstitial nephritis in VL do not always correlate to treatment, as similar profiles have been observed in patients undergoing different therapeutic regimens or even in those not undergoing any treatment^53^.

The precise mechanisms through which VL can induce kidney injury are not fully understood. Notably, the presence of *Leishmania* in renal tissue^40,75^, specifically in the glomeruli^52^ has been reported. In connection with this, large and low-soluble complexes were observed in the glomeruli of patients with VL^76^. Considering that AKI is an important clinical feature of VL, we hypothesized that this clinical condition could be related to the high levels of anti-*Leishmania* Igs and CIC, suggesting their deposition on renal tissue. Thus, we aimed to evaluate the dynamics of CIC in patients with VL and explore the association between CIC and the biological markers of AKI.

## Results

### Clinical and demographic characteristics of patients with VL

Fourteen patients with VL were enrolled in this study. Table 1 displays the main clinical and demographic characteristics of this cohort, which consisted predominantly of men (11/14; 78%), with a median age of 41.5 years (Inter quartile range 31.0–46.5).

**Table 1:** Clinical and demographic characteristics of VL patients.

Patient ID	Age	Sex	Staging of AKI	VL treatment	Cumulative Amph. B dose	Associated conditions
VL01	28	M	1	D-Amph. B 50 mg/day per 12 days followed by L-Amph. B 20 mg/Kg	28.5 mg/Kg	No information
VL02	19	M	2	L-Amph. B 20 mg/Kg	20 mg/Kg	No information
VL03	48	M	2	D-Amph. B 50 mg/day per 3 days followed by L-Amph. B 20 mg/Kg	21.7 mg/Kg	Undetermined form of Chagas Disease
VL04	32	M	1	D-Amph. B 50 mg/day per 4 days followed by L-Amph. B 20 mg/Kg	22 mg/Kg	Virchowian hanseniasis, ADHD, Development delay—infantilized behavior
VL05	45	M	Non-AKI	D-Amph. B—1000 mg	No exact dose information available	Dyslipidemia
VL06	38	M	1	Amph. B Lipid Complex 20 mg/Kg	20 mg/Kg	Hypertension, Diabetes mellitus type 2, Metabolic syndrome
VL07	24	M	1	Amph. B Lipid Complex 40 mg/Kg	40 mg/Kg	No information
VL08	45	M	2	Amph. B Lipid Complex 20 mg/Kg	20 mg/Kg	Hypertension
VL10	62	M	3	Amph. B Lipid Complex 25 mg/Kg	25 mg/Kg	Chagas heart disease
VL11	61	M	3	Amph. B Lipid Complex 200 mg/day per 4 days followed by L-Amph. B 20 mg/Kg	20 mg/Kg	Schistosomiasis and gonorrhea
VL12	32	F	1	D-Amph. B 150 mg followed by L-Amph. B 20 mg/Kg	23 mg/Kg	No information
VL13	33	F	Non-AKI	Meglumine antimoniate 20 mg Sb^+5^/Kg/day for 3 days followed by L-Amph. B 20 mg/Kg	20 mg/Kg	No information
VL14	46	M	1	L-Amph. B 20 mg/Kg	20 mg/Kg	Hypertension
VL15	45	F	1	L-Amph. B 40 mg/Kg	40 mg/Kg	Pulmonary hypertension, hypothyroidism

ADHD, attention deficit hyperactivity disorder; AKI, acute kidney injury; Amph. B, Amphotericin B; D-Amph. B, amphotericin B deoxycholate; F, female; ID, identification; L-Amph. B, liposomal amphotericin B; M, male; sCr, serum creatinine; VL, visceral leishmaniasis.

VL treatment was prescribed according to the Brazilian Minister of Health guidelines; however, a high changeability was observed based on clinical considerations. Four patients started VL treatment with amphotericin B deoxycholate (D-Amph. B) and required the switch to liposomal formulation due to adverse events. Similarly, one patient started treatment with lipid complex and another one with meglumine antimoniate, switching to liposomal amphotericin B (L-Amph. B) due to adverse effects. One patient completed VL treatment using D-Amph. B. Moreover, seven patients completed VL treatment using amphotericin B lipid formulations. The daily amphotericin B dose ranged from 1.4 to 5.0 mg/Kg, with a median of 2.5 mg/Kg. At the end of 12 months follow-up, all patients had achieved complete remission of VL symptoms and were considered cured.

The associated conditions reported was hypertension (3/14), diabetes mellitus type 2 (1/14), dyslipidemia (1/14), pulmonary hypertension (1/14), hypothyroidism (1/14), attention deficit hyperactivity disorder (1/14), development delay (1/14), Virchowian hanseniasis (1/14), undetermined form of Chagas disease (1/14), Chagas heart disease (1/14), schistosomiasis (1/14), and gonorrhea (1/14).

### High levels of CIC and anti-*Leishmania* immunoglobulins in patients with VL

**Figure 1:**
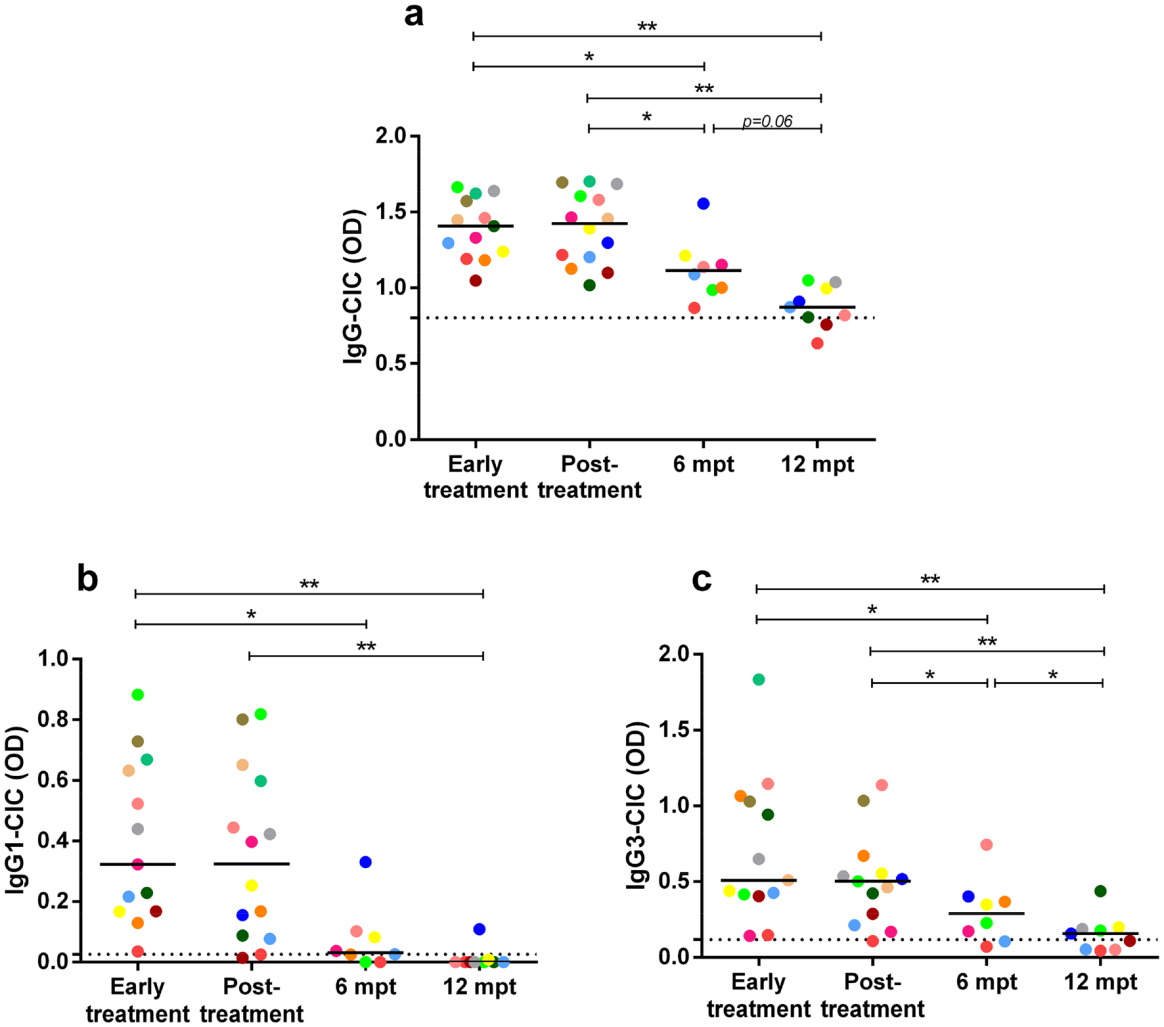
CIC levels in patients with VL throughout the clinical follow-up. IgG- (**a**), IgG1- (**b**), and IgG3- (**c**) containing immune complexes. Each symbol represents a patient with VL. Each color represents the same patient in different phases of clinical follow-up. The horizontal bars represent the median values of each group. The dashed line represents the median value of healthy individuals. CIC, circulating immune complexes. Mpt, months post-treatment. OD, optical density. Asterisks denote significant differences between the phases of clinical follow-up: *p < 0.05. **p < 0.01.

**Figure 2:**
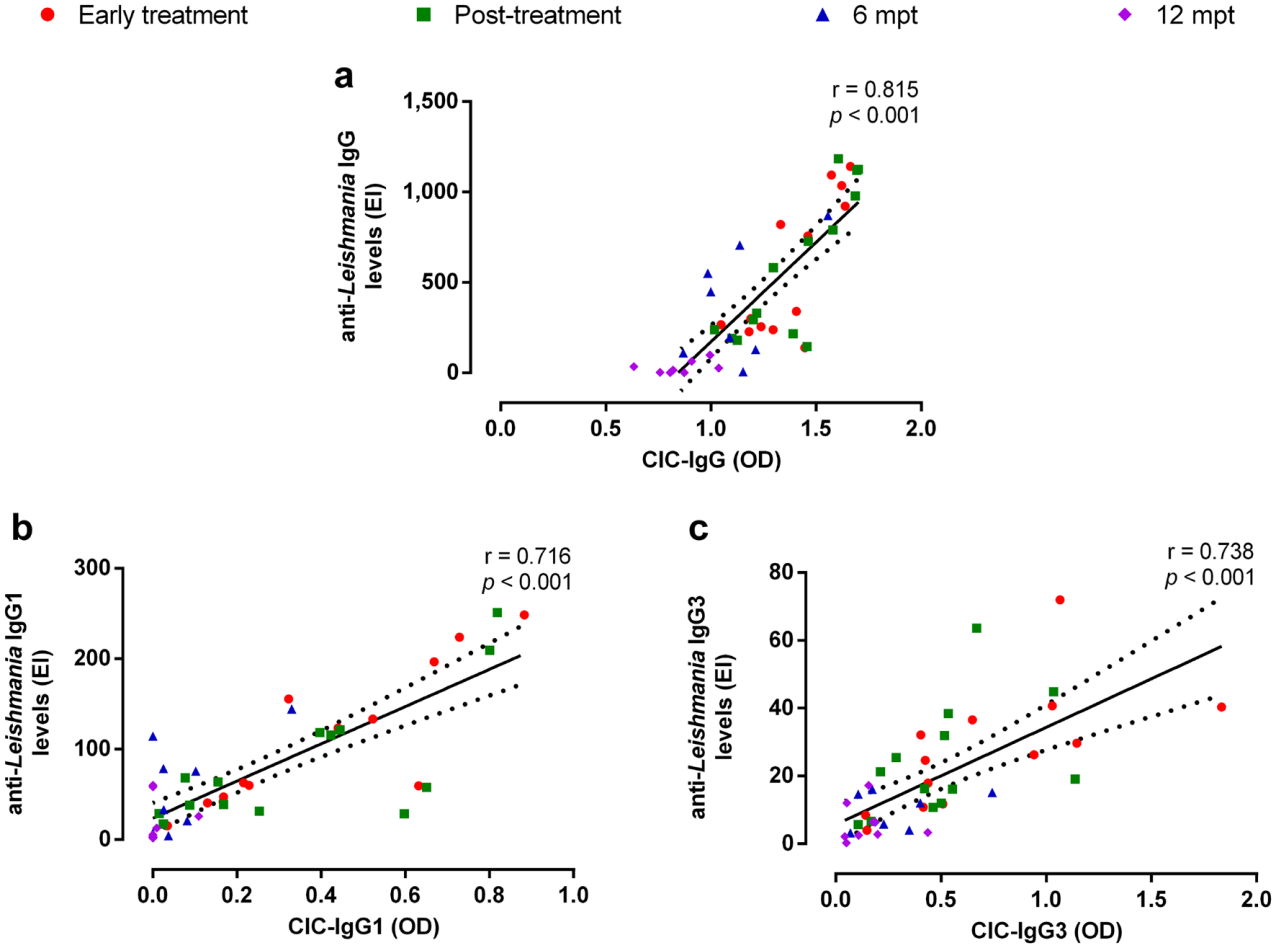
Correlation between CIC levels and anti-*Leishmania* Igs levels throughout the clinical follow-up. Positive correlation of IgG (**a**, Spearman correlation, r = 0.815, *p* < 0.001), IgG1 (**b**, Spearman correlation, r = 0.716, *p* < 0.001), and IgG3 (**c**, Spearman correlation, r = 0.738, *p* < 0.001). CIC: circulating immune complexes. EI: ELISA index. Mpt: months post-treatment. OD: optical density.

At early stages of clinical follow-up, patients with VL presented elevated levels of CIC comprised of IgG (Fig. 1a), IgG1 (Fig. 1b), or IgG3 (Fig. 1c) subclasses when compared to HC (*p* < 0.05). All CIC levels decreased throughout the clinical follow-up, being significantly lower at 6 mpt and 12 mpt when compared to the values observed at early treatment. Nevertheless, IgG- or IgG3- containing CIC remained significantly elevated when compared to HC up to 6 mpt (*p* < 0.05). Notably, the same profile was observed in anti-*Leishmania* IgG and subclasses of immunoglobulins (Supplementary Fig. S1). Interestingly, levels of IgG- and subclasses- containing CIC were positively correlated with anti-*Leishmania* Igs (Fig. 2; *p* < 0.001), which reinforced that specific Igs may be contributing to the CIC formation.

### AKI was observed in hospitalized patients with VL

**Figure 3:**
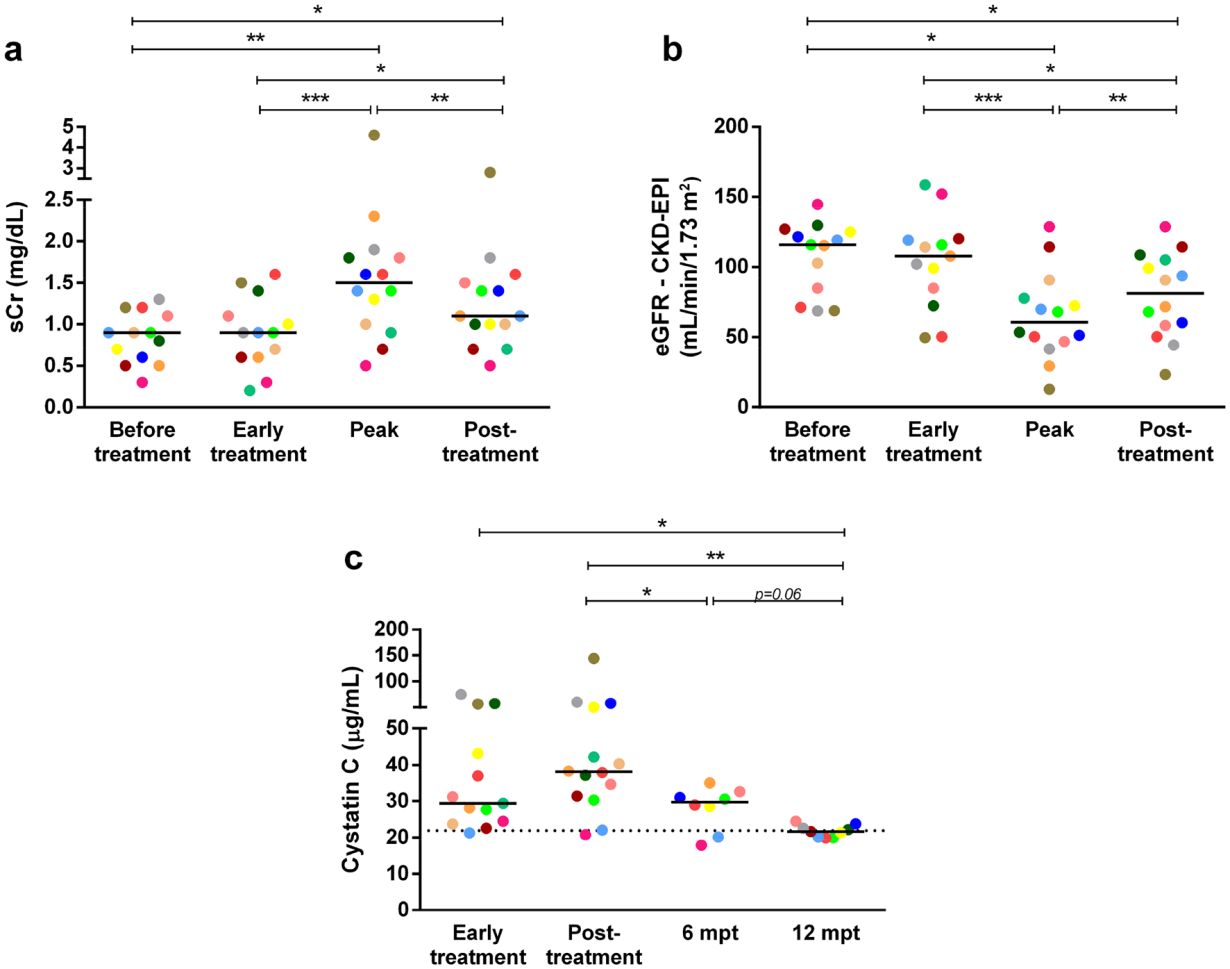
Markers of renal function in patients with VL throughout the clinical follow-up. sCr (**a**) and eGFR rate by CKD-EPI equation (**b**) during hospitalization. Peak demonstrates the highest value of sCr recorded during the hospitalization period. The temporal distribution of this point regarding anti-*Leishmania* treatment is demonstrated in Supplementary Fig. S2. Cystatin C plasmatic levels (**c**) during the prospective follow-up. Each symbol represents a patient with VL. Each color represents the same patient in the different phases of clinical follow-up. The horizontal bars represent the median values of each group. The dashed line represents the median value of healthy individuals. eGFR, estimated glomerular filtration rate. Mpt, months post-treatment. sCr, serum creatinine. Asterisks denote significant differences between the phases of clinical follow-up: *p < 0.05. **p < 0.01. ***p < 0.001.

The two laboratory markers chosen as indicators for renal function, namely sCr and eGFR revealed aggravation of renal functions during the prospective follow-up. Thus, patients with VL had increased sCr levels (Fig. 3a), which were significantly higher when compared to the sCr recorded before and even after early treatment (*p* < 0.05). The temporal distribution of peak sCr regarding anti-*Leishmania* treatment (Supplementary Fig. S2) indicated that the aggravation occurred mainly during the treatment phase (85.7%; 12 of 14 patients). While the sCr levels exhibited a reduction in the post-treatment phase (*p* < 0.05), the aforementioned levels remained elevated in comparison to the early stages of follow-up (Fig. 3a). Additionally, the eGFR rate calculated using the CKD-EPI equation revealed an impairment in kidney function in most of the patients with VL (Fig. 3b). Considering the sCr levels, 12 (85%) patients met the AKI criteria according to KDIGO 2012 guidelines (Table 1). Although few patients (2/12) reached level 3 renal dysfunction, the majority experienced mild (7/12) to moderate intensity (3/12).

Concomitantly, higher levels of cystatin C were observed in patients with VL at early treatment when compared to HC (*p* < 0.0005). The aforementioned levels remained elevated up to 6 mpt (*p* < 0.05), reaching values similar to HC only at 12 mpt (Fig. 3c). Interestingly, cystatin C levels were positively correlated with sCr (Spearman correlation, r = 0.586, *p* = 0.001) and negatively correlated with eGFR (Spearman correlation, r = -0.668, *p* < 0.001) (Supplementary Fig. S3).

Urinalysis data obtained from 7 patients with VL revealed abnormalities such as hematuria (5/7), pyuria (6/7), proteinuria (4/7), and granular casts (4/7) before or at early treatment visits.

### Kidney impairment in visceral leishmaniasis was associated with high levels of anti-*Leishmania* immunoglobulins and CIC

To explore the relationship between immune response parameters and renal dysfunction, cystatin C evaluation was performed concurrently with the Igs and CIC measurements.

**Figure 4:**
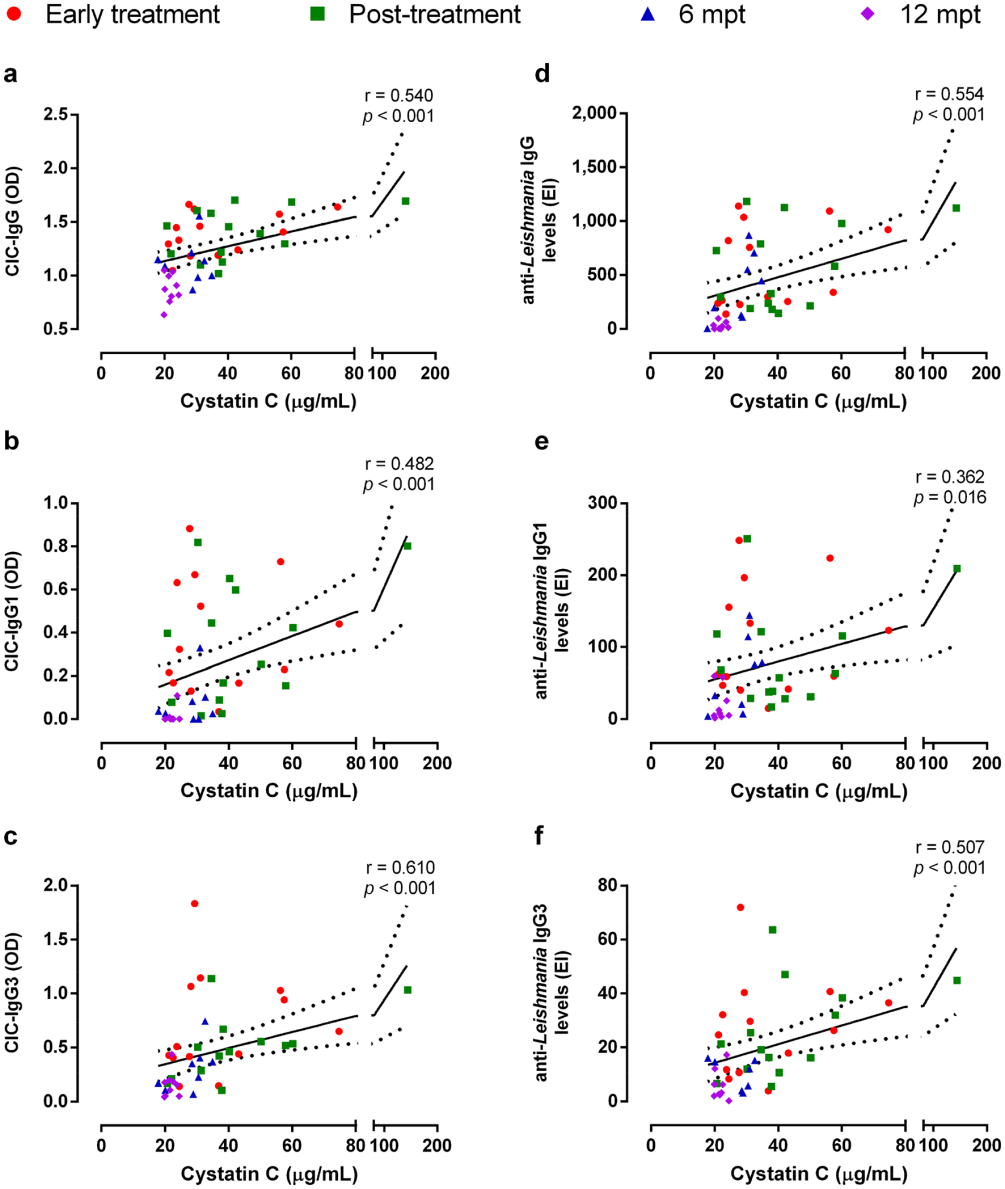
Correlation between cystatin C and CIC or anti-*Leishmania* Igs throughout the clinical follow-up. Positive correlation between cystatin C and IgG- (**a**, Spearman correlation, r = 0.540, *p* < 0.001), IgG1- (**b**, Spearman correlation, r = 0.482, *p* < 0.001) and IgG3- (**c**, Spearman correlation, r = 0.610, *p* < 0.001) CIC. Positive correlation between cystatin C and levels of anti-*Leishmania* IgG (**d**, Spearman correlation, r = 0.554, *p* < 0.001), IgG1 (**e**, Spearman correlation, r = 0.362, *p* < 0.016) and IgG3 (**f**, Spearman correlation, r = 0.507, *p* < 0.001). CIC, circulating immune complexes.

Remarkably, plasmatic cystatin C levels were positively correlated with CIC containing IgG (Fig. 4a), IgG1 (Fig. 4b) and IgG3 (Fig. 4c) (Spearman correlation—IgG: r = 0.54, *p* < 0.001; IgG1: r = 0.48, *p* < 0.001; IgG3: r = 0.61, *p* < 0.001), as well as with anti-*Leishmania* IgG (Fig. 4d), IgG1 (Fig. 4e) or IgG3 (Fig. 4f) levels (Spearman correlations—IgG: r = 0.55, *p* < 0.001; IgG1: r = 0.36, *p* = 0.01; IgG3: r = 0.50, *p* < 0.001), suggesting that high levels of CIC or specific Igs may be involved in the AKI observed in patients with VL. Most importantly, multivariate analysis demonstrated that CIC containing IgG only or CIC-IgG and anti-*Leishmania* IgG3 were predictors for cystatin C levels in patients with VL (Table 2).

**Table 2:** Multivariate linear regression analysis to evaluate the association between cystatin C levels and independent variables in VL patients.

Independent variables	Dependent variable			
	Cystatin C			
	Correlation coefficient	Standard error	R^2^	*p*
1—IgG containing CIC	0.555	0.155	0.308	< 0.0001
2—IgG containing CIC anti-*Leishmania* IgG3	0.626	0.147	0.392	< 0.0001

### Elevated levels of complement system activation products in patients with VL and AKI

**Figure 5:**
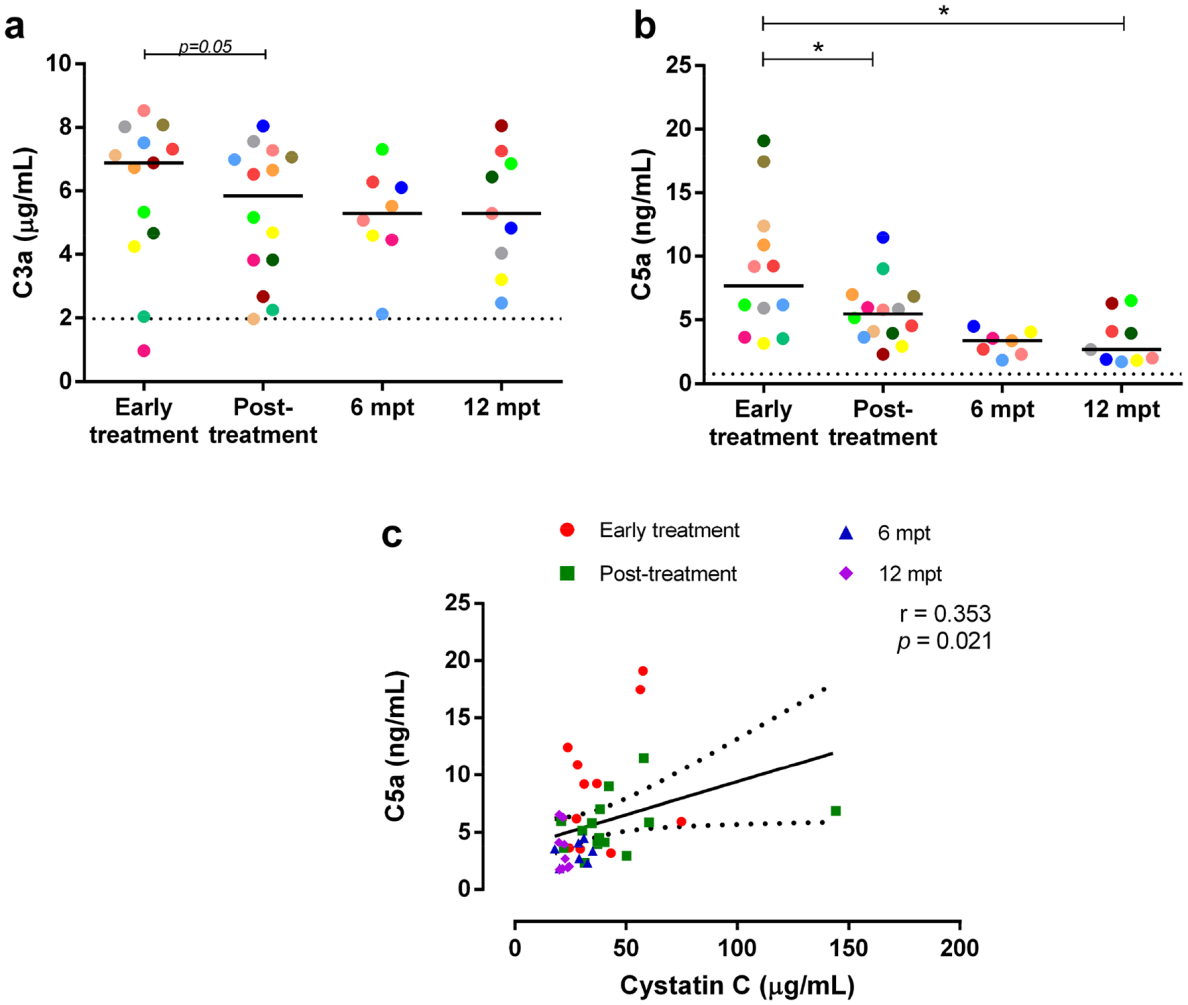
Levels of complement anaphylatoxins C3a and C5a in patients with VL throughout the clinical follow-up. C3a (**a**) and C5a (**b**). Each symbol represents a patient with VL. Each color represents the same patient in the different phases of clinical follow-up. The horizontal bars represent the median values of each group. The dashed line represents the median value of healthy individuals. Mpt, months post-treatment. Asterisks denote significant differences between the phases of clinical follow-up: *p < 0.05. Positive correlation between cystatin C and C5a throughout clinical follow-up (**c**, Spearman correlation, r = 0.353,* p* = 0.021).

Considering that high production of CIC leads to the activation of the complement system as an important clearance mechanism, we evaluated the levels of anaphylatoxins C3a and C5a as products of this activation cascade. Subsequently, we examined whether these molecules exhibited any correlation with kidney injury. Remarkably, patients with VL presented high levels of C3a (Fig. 5a) throughout the clinical follow-up, with a slight decrease at post-treatment. The levels were higher than those observed in HC, although no significant difference was observed.

Concomitantly, high levels of C5a (Fig. 5b) were observed in patients with VL at the initial stages of clinical follow-up when compared to HC (*p* < 0.05), despite a significant decrease observed in post-treatment visit. The levels decreased only at 6 mpt and 12 mpt, reaching values similar to those observed for HC. Interestingly, C5a levels were positively correlated with cystatin C (Spearman correlation, r = 0.353,* p* = 0.021) (Fig. 5c) and with CIC containing IgG, IgG1, or IgG3, as well as anti-*Leishmania* Igs levels (Supplementary Fig. S4) (Spearman correlations, *p* < 0.02). The data mentioned above suggest that complement system activation driven by CIC may be involved in AKI observed in patients with VL.

### Association between AKI and VL treatment

**Figure 6:**
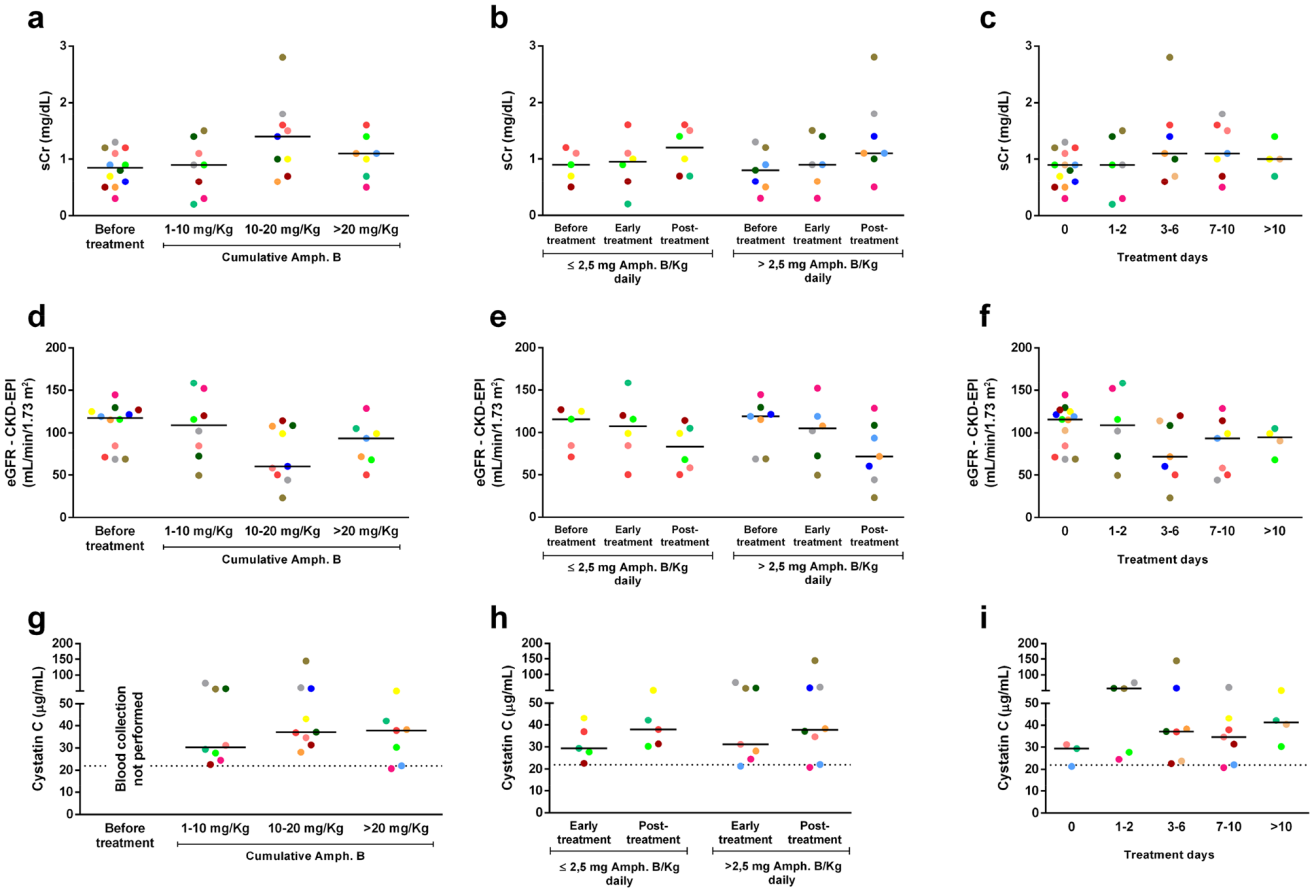
Markers of renal function in patients with VL throughout the anti-*Leishmania* treatment. sCr (**a**–**c**), eGFR (**d**–**f**), and cystatin C (**g**–**i**) levels according to the cumulative amphotericin B dose, daily amphotericin B dose, or treatment days, respectively. Each symbol represents a patient with VL. Each color represents the same patient in the different phases of anti-*Leishmania* treatment. The horizontal bars represent the median values of each group. Amph. B, Amphotericin B. sCr, serum creatinine. eGFR, estimated glomerular filtration rate.

To better understand whether the impairment in renal function could be related to VL treatment, we stratified the patients according to daily and cumulative doses of amphotericin B as well as treatment days (Fig. 6). No significant changes in sCr, eGFR, or cystatin C levels were observed during the amphotericin use, suggesting that AKI in our cohort does not seem to be drug-induced.

## Discussion

Renal involvement in visceral leishmaniasis has been recognized for many years, with several studies reporting the occurrence of urinary abnormalities^6,48–50^, tubulointerstitial nephritis, GN, and AKI^49–56^. Nonetheless, the mechanisms by which VL can impair kidney function are not fully understood. This study prospectively evaluated the involvement of anti-*Leishmania* Igs and CIC in the occurrence of AKI in a cohort of hospitalized patients with VL under treatment from Minas Gerais.

Previous studies in Ethiopia^50^ and Brazil^8,54, 55, 59^ addressing AKI in VL identified several risk factors such as male sex, advanced age, jaundice, hyperglobulinemia, hypoalbuminemia, and HIV or other secondary infections^8,50, 59^. The use of amphotericin B or other nephrotoxic drugs for VL treatment, alongside pre-renal factors such as hypotension and fluid loss, as well as the presence of parasites in renal tissue, have been implicated in renal damage in VL^40,50, 52, 55, 59, 64–67, 75^. However, no consensus on the definitive factors involved in the occurrence of AKI in VL remains.

Similarly, data on AKI prevalence are inconsistent. A previous Brazilian study in Ceará reported 26.3% (15 of 57 patients) of AKI in patients admitted at a reference hospital^54^. Subsequently, a prevalence of 33.9% (76 of 224 patients)^59^ and 46% (23 of 82 patients)^55^ was reported in adult cohorts recruited at the same hospital. A similar rate (45.9%; 67 of 146 patients) of AKI was also demonstrated in children with VL^8^, and 17.4% (52 of 298 patients) of AKI was reported in an Ethiopian cohort^50^. Herein, we equally demonstrated a high prevalence of kidney injury in patients with VL, accounting for 85.7% (12 of 14) of patients. This prevalence was greater than that reported previously by other groups, which may be attributed to different criteria used to determine AKI, sample size, or even severity of patients, as kidney failure is an important criterion to predict VL death^5^.

Notably, these studies evaluated renal function using sCr levels, since this is commonly the endogenous marker used in clinical practice and for staging AKI according to KDIGO guidelines. Nonetheless, the aforementioned levels can be affected by diet, muscular mass, age, sex, drugs, and other endogenous substances such as bilirubin^77–79^. Therefore, cystatin C has been considered an alternative and important renal injury biomarker as it is not affected by the factors mentioned^77–81^. Additionally, cystatin C presents higher sensitivity when compared to sCr, since its levels exhibit elevation even in cases of mild renal function impairment^77^. Here, we concurrently evaluated the renal function through plasmatic levels of cystatin C and sCr in patients with VL, which indicated an increase in cystatin C from early treatment visit, while sCr, and in turn, eGFR, remained unchanged in the same period. Thus, our data reinforced previous reports^51,82^ that cystatin C is a superior marker of renal function than sCr due to better sensitivity and consistency. Despite some reports of changing inflammation^83,84^, this cohort did not exhibit a positive relationship between cystatin C and inflammatory markers, such as IL-1β, IL-6, TNF, and IFN-γ in bivariate (Supplementary Table S1) and multivariate analysis, supporting the use of cystatin C as kidney injury biomarker even in infectious diseases^85–87^.

Additionally, the long-term assessment of cystatin C levels displays that kidney injury in VL context has a transitory feature with mild to moderate intensity, in accordance with previous reports^50^. This underscores the necessity of more sensitive biomarkers to assess renal function in patients with VL, which could lead to early detection of kidney impairment. In this context, elevated levels of serum neutrophil gelatinase-associated lipocalin were also observed in patients with VL who developed AKI^55^, thus emphasizing the potential of these molecules as promising biomarkers to guide the clinical management of VL.

Nonetheless, only a limited number of studies have sought to understand whether the immune response to VL and its immunopathogenic consequences might be implicated in this acute renal impairment. Considering the extent of immune activation in VL, particularly the humoral response, we posit that an underlying cause of kidney damage could plausibly be of immunological origin. Consequently, the excessive production of Ig, coupled with the subsequent formation of CIC that can aggregate and preferentially accumulate on endothelium cells within high-pressure vessels, assumes a pivotal role in kidney inflammation through activation of the complement system, and destruction of nephron units^88^.

As anticipated, patients with VL had elevated levels of CIC containing IgG, IgG1, and IgG3. Interestingly, CIC was positively correlated with the levels of specific immunoglobulins, indicating the presence of parasite antigens in the CIC composition, as previously reported^33,34^. A positive correlation between the levels of cystatin C and CIC (IgG-, IgG1- and IgG3- containing) or *Leishmania*-specific Igs (IgG, IgG1, and IgG3) was observed in patients with VL. Additionally, the multivariate analysis revealed that CIC containing IgG or CIC-IgG and anti-*Leishmania* IgG3 were predictors for cystatin C levels, suggesting an immunological nature of VL renal damage, which corroborates with the findings reported by Elshafie et al.^60^.

Concomitantly, our findings align with the established knowledge regarding the relationship between CIC and tissue injury in models of autoimmunity and other infectious diseases^89,90^. In VL, earlier reports underscored the occurrence of GN with Igs deposits in both animal models and human disease^39,46, 47, 76^. Although the direct assessment of in situ CIC was not feasible in this study, a significant proportion of patients whose urinalysis data were evaluated exhibited indications of glomerular inflammation, as evidenced by hematuria^91,92^, pyuria, proteinuria, and granular casts– indicative of an immune complex-mediated GN.

Emphasizing CIC formation as an inherent and constant process, typically accompanied by subsequent mechanisms of inactivation and elimination, in which the activation of the complement system assumes a central role is important^35^. Notably, in our cohort, high serum levels of C3a and C5a were observed. Interestingly, CIC containing IgG, IgG1, or IgG3, and specific Igs levels are positively correlated with C5a levels, which reinforced that high levels of CIC are associated with activation of the complement system. The activation of the complement system stands as a key mechanism involved in GN mediated by immune complexes^89^. Considering this and the fact that VL itself may facilitate CIC deposits in several ways, the positive correlation between C5a and cystatin C levels implies the potential involvement of the complement system activation in renal damage among patients with VL. Furthermore, the importance of C5a and its receptor (C5aR) in kidney diseases has been described in animal models of lethal C3 glomerulopathy^93^, ischemia-reperfusion^94,95^, lupus nephritis^96^, immunoglobulin A nephropathy^97^, rhabdomyolysis-induced AKI^98^, diabetic kidney disease^99^ and in human mesangial proliferative GN^100^. This involvement primarily manifests through a C5a-mediated proinflammatory response and apoptosis and may be occurring in VL.

In concurrence with this, amphotericin B, the first-line treatment for VL according to the Brazilian Minister of Health guidelines, is usually associated with severe side effects, such as nephrotoxicity and AKI^8,54, 59^. Nonetheless, the severity of these side effects depends on the formulation used during treatment. Lipidic formulations typically have more tolerability and provide a safer and more effective treatment, albeit still warranting a careful monitoring^73,74^. Corroborating with the aforementioned data, five patients in this study initially received D-Amph. B, while only one patient received meglumine antimoniate as the initial choice for VL treatment. The majority of the patients (4 out of 5 with D-Amph. B and meglumine antimoniate) presented complications during VL treatment, predominantly of renal or hepatic nature. Given this, L-Amph. B was prioritized as a replacement owing to its safety. It may explain the higher prevalence of AKI in patients with VL observed in our study as compared to the data reported by other groups.

Furthermore, because of L-Amph. B is the preferred treatment for patients with VL with severe conditions, such as HIV coinfection or kidney impairment, the occurrence of AKI could be mistakenly overestimated when associated with this treatment. Thus, patients with VL concomitantly infected with HIV or with a previous impaired kidney function were excluded from this evaluation. In addition, the patients were stratified according to daily and cumulative doses of amphotericin B and days of treatment to assess their influence on renal injury. Interestingly, by previous reports^8,101^, no significant changes in sCr, eGFR, or cystatin C levels were observed during the amphotericin B use in our cohort. Nevertheless, this does not eliminate the potential impact of these factors on the observed phenomenon, particularly since we lack a comprehensive study design for this purpose, and the relationship between amphotericin B formulations and AKI was not investigated in this study.

Here, we seek to highlight that other factors, such as parameters of the immune response in VL potentially contribute to renal injury. Thus, AKI in patients with VL was associated with high levels of specific Igs, CIC, and C5a. Concomitantly, despite no relationship between amphotericin B use and renal function impairment in our cohort, we believe that the treatment may be an important cofactor since it can release parasitic antigens into the circulation^51,102^, favoring the formation of immune complexes and complement system activation.

Despite the limitations of our study, including its relatively small sample size, absence of urine markers evaluation, or the deposition of immunocomplexes in situ, the findings presented herein underscore an additional mechanism to join other evidence for the full understanding of VL-related renal dysfunction. Our findings demonstrated a significant issue that warrants comprehensive exploration in a subsequent multicentric study involving a larger number of patients, intrinsic differences to the parasite, population, clinical severity, and therapeutic interventions.

In summary, AKI is an important issue in VL, being associated with morbidity and mortality^54,55^. The occurrence of AKI has a multifactorial feature, in which comorbidities, hemodynamic abnormalities, VL treatment, or use of nephrotoxic drugs as well as levels of immunocomplexes and immunoglobulins and complement system activation may be associated with nephrotoxicity^8,55, 59, 64–67^. Given this scenario, the need for a multicenter study is evident, with standardized and well-controlled clinical and immunological analyses to determine the actual occurrence of AKI in patients with VL. This approach will facilitate a more precise definition of the factors involved in kidney disease associated with VL. Moreover, our data reinforces the importance of careful monitoring of renal functions in patients with VL, employing markers with high sensitivity. This early detection of VL-associated kidney injury is essential for averting complications and guiding the clinical management of the disease.

## Methods

### Casuistic and study design

Fourteen patients with VL were consecutively recruited from an infectious disease referral hospital in Belo Horizonte, Minas Gerais State, Brazil (Hospital Eduardo de Menezes—Fundação Hospitalar do Estado de Minas Gerais/HEM-FHEMIG) between May 2018 and November 2020. The cohort was prospectively followed for 12 months, in which patients were evaluated through four scheduled visits: early treatment; immediately post-treatment; 6 months post-treatment (mpt); and 12 mpt. Blood samples were collected at each visit to evaluate the anti-*Leishmania* IgG, IgG1, and IgG3, as well as the immune complexes levels. Moreover, 22 healthy participants (HC) were included as controls.

All recruited patients manifest clinical signs and symptoms compatible with VL and their laboratorial diagnostic confirmation was established using parasitological tests (e.g., direct smear, culture, or polymerase chain reaction of bone marrow aspirate) or serology. In addition, the study included patients aged 18 years or older, not pregnant, and human immunodeficiency virus (HIV) seronegative, regardless of gender. Patients with a history of chronic kidney disease were excluded. Additionally, VL therapy was defined on an individual basis according to the Brazilian Minister of Health guidelines and local drug availability. The demographic, clinical, and laboratory information, in addition to treatment details, and outcomes were extracted from medical records.

### Anti-*Leishmania* immunoglobulin assessment

An enzyme-linked immunoassay (ELISA) was performed as previously described^13^. Briefly, soluble antigens (40 µg/mL) from *L.* *infantum* promastigotes (MHOM⁄BR⁄1974⁄PP75) were used to coat a polystyrene flat-bottom microtiter plate (Nunc-Immuno, Roskilde, Denmark). The plates were washed with phosphate buffer saline (PBS) containing 0.05% Tween-20 (PBS-T), and PBS containing 10% fetal bovine serum was used as a blocking solution. The plasma samples were diluted as follows: 1:10000 to IgG, 1:2000 to IgG1, 1:200 to IgG3. Subsequently, diluted peroxidase-conjugated monoclonal antibody anti-human IgG (1:1000; Invitrogen, San Francisco, CA, USA), IgG1 (1:500), and IgG3 (1:400) (Zymed Laboratories Inc., San Francisco, CA, USA) were used. The absorbance was measured using a Benchmark microplate reader (Bio-Rad Laboratories, Hercules, CA, USA) at 492 nm. The results were expressed as an ELISA index (EI), which is based on the division of the average optical density (OD) of the duplicates of the patient samples, by the average OD obtained from the negative controls.

### Circulating immune complexes (CIC) assessment

An ELISA assay was performed to assess the CIC levels. Briefly, complement C3b monoclonal antibody (2,5 µg/mL; Invitrogen, San Francisco, CA, USA) was used to coat a polystyrene flat-bottom microtiter plate (Nunc-Immuno, Roskilde, Denmark). The plates were washed with PBS-T and PBS containing 1% bovine serum albumin was used to block the plate. Plasma samples from patients with VL were diluted at 1:50. Then, diluted peroxidase-conjugated monoclonal antibody anti-human IgG (1:1000; Invitrogen, San Francisco, CA, USA), IgG1 and IgG3 (1:500; Invitrogen, San Francisco, CA, USA) were used. The OD was determined using the microplate reader Loccus LMR-96 using 450 nm as the primary and 630 nm as the reference wavelength. The results were expressed as OD, calculated as the mean of duplicates of patient samples.

### Evaluation of renal function markers

At each time of interest, before and after treatment, the eGFR was calculated using the chronic kidney disease epidemiology collaboration (CKD-EPI) equation. The peak serum creatinine (sCr) (highest sCr value recorded during the hospitalization period) was used to stage AKI by the Kidney Disease—Improving Global Outcomes (KDIGO) Clinical Practice Guideline of 2012^103^ as follows: Stage 1 includes patients with an increase in sCr ≥ 0.3 mg/dL within 48 h or 1.5–1.9 times baseline; Stage 2 includes patients with an increase in sCr ≥ 2.0–2.9 times baseline; and Stage 3 includes patients with an increase in sCr ≥ 4.0 mg/dL or ≥ 3.0 times baseline. The sCr levels obtained in the “before treatment” visit were considered as the baseline values.

Moreover, the VL plasma samples were used to quantify the cystatin C levels using a commercial kit (Millipore, Saint Louis, MO, USA), according to the manufacturer’s recommendations. A seven-point standard curve and the plasma samples were quantified in duplicate. Furthermore, the OD was determined using the microplate reader Loccus LMR-96 at 450 nm. The results were expressed in micrograms per milliliter (µg/mL), and the minimum detection limit was 300 picograms per milliliter (pg/mL).

### Quantification of inflammatory markers of complement system activation C3a and C5a

The levels of C3a and C5a anaphylatoxins were quantified in plasma samples using a commercial kit (Invitrogen, San Francisco, CA, USA), according to the manufacturer’s recommendations. A seven-point standard curve and the plasma samples were quantified in duplicate. The OD was determined using the microplate reader Loccus LMR-96 using 450 nm as the primary and 630 nm as the reference wavelength. The results of C3a and C5a were expressed in micrograms per milliliter (µg/mL) and nanograms per milliliter (ng/mL), respectively. The minimum detection limit for C3a was 0.14 ng/mL and 0.005 ng/mL for C5a.

### Statistical analysis

Comparisons between patients with VL and healthy controls were performed using the unpaired and non-parametric Mann–Whitney U test. Wilcoxon tests were used for paired variables with skewed distributions, which involved the same individual at different time points. Spearman’s test was used for bivariate correlation analysis. The quantitative results were expressed as median values. The statistical analyses were performed using GraphPad Prism software (version 6.0, San Diego, CA, USA).

A multivariate linear regression analysis using SPSS software version 21 (IBM, Armonk, NY, USA) was performed to determine the influence of intervenient factors on the cystatin C levels. IgG-, IgG1- and IgG3- containing CIC, anti-*Leishmania* Igs, C3a, C5a, as well as inflammatory cytokines—IL-1 beta (IL-1β), IL-6, tumor necrosis factor (TNF), and Interferon-gamma (IFN-γ) were considered as independent variables. The dependent variable was logarithmized to fit the premise of data normality required by linear regression. To verify the global significance of the regression model, the analysis of variance and Fisher's test analysis were performed. The Durbin–Watson test was used to determine if an autocorrelation in the residuals of the regression models exists. Differences were considered statistically significant when the *p-value* is < 0.05.

### Ethics approval and consent to participate

This study has been reviewed and approved by the Ethics Committee of Hospital Eduardo de Menezes, Instituto Rene Rachou, and Instituto Oswaldo Cruz—Fundação Oswaldo Cruz. The patients/participants provided their written informed consent to participate in this study. All experiments were performed in accordance with relevant named guidelines and regulations.

### Supplementary Information

Supplementary Information.

## Data Availability

The datasets generated and/or analyzed during the current study are not publicly available due individual privacy of patients could be compromised but are available from the corresponding author on reasonable request.
