# Supplementary material for: A link between circulating immune complexes and acute kidney injury in human visceral leishmaniasis
Source: Sci Rep. 2024 Apr 30;14:9870. doi: 10.1038/s41598-024-60209-0 (PMC11059367; doi:10.1038/s41598-024-60209-0)
Supplement: Supplementary file 1 — Supplementary Information. [file 41598_2024_60209_MOESM1_ESM.doc]

**A link between circulating immune complexes and acute kidney injury in human visceral leishmaniasis**

**Gabriela Corrêa-Castro^1,2^; Maria Luciana Silva-Freitas^1^; Ludmila de Paula^3^; Leonardo Soares Pereira^3^; Maria Rita Teixeira Dutra^3^; Hermano Gomes Albuquerque^4^; Glaucia Cota^5^; Caroline de Azevedo Martins^6^; Alda Maria Da-Cruz^1,7,8,9^; Adriano Gomes-Silva^1,10^; Joanna Reis Santos-Oliveira^1,2,9 *^**

**Supplementary information**


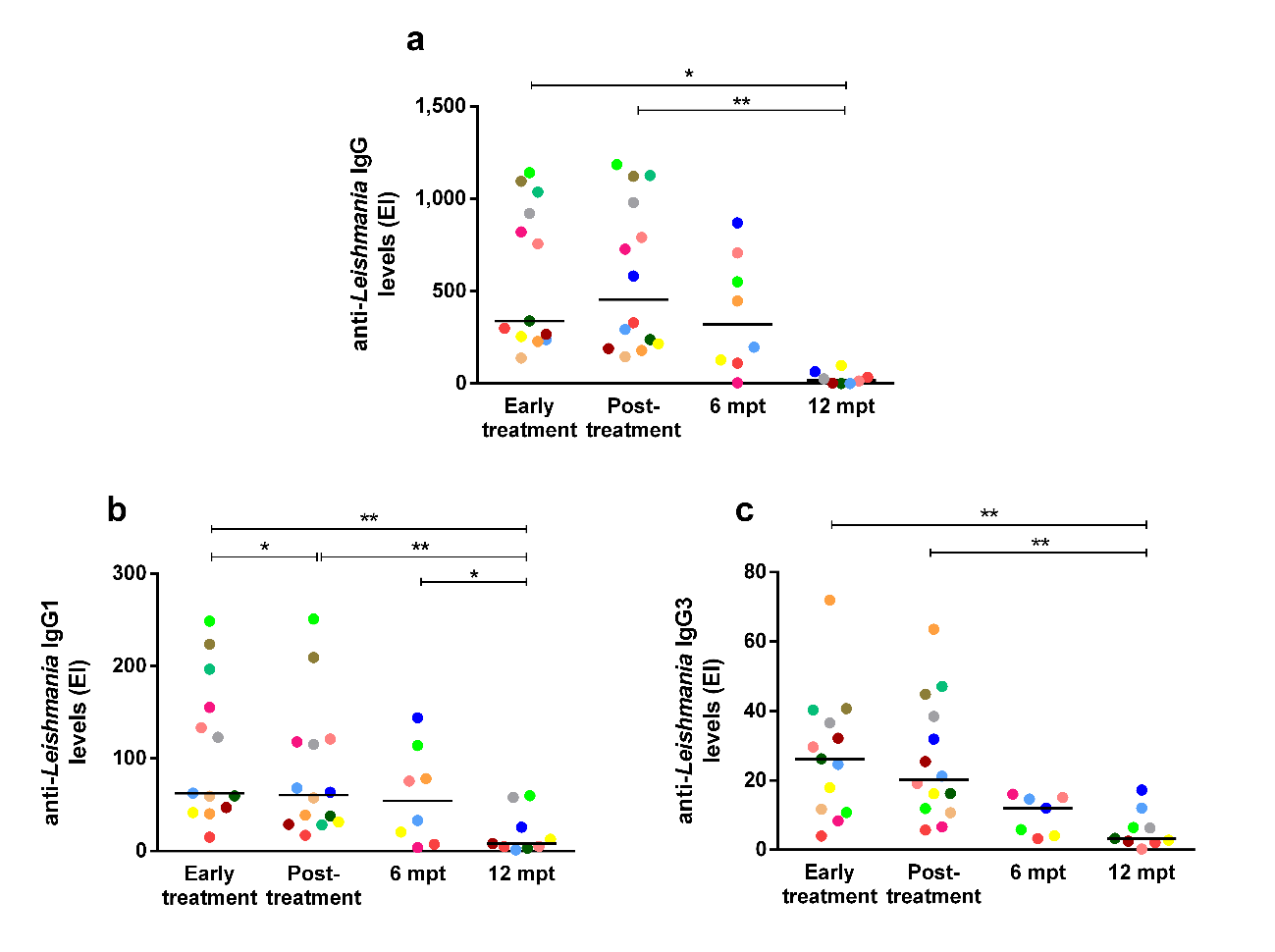


**Supplementary Figure S1: Anti-*Leishmania* Igs levels in patients with VL throughout the clinical follow-up.** Titers of IgG (A), IgG1 (B) and IgG3 (C). Each symbol represents a patient with VL. Each color represents the same patient in the different phases of clinical follow-up. The horizontal bars represent the median values of each group. EI: ELISA Index. Mpt: months post-treatment. Asterisks denote significant differences between the phases of clinical follow-up: **p* < 0.05. ***p* < 0.01.


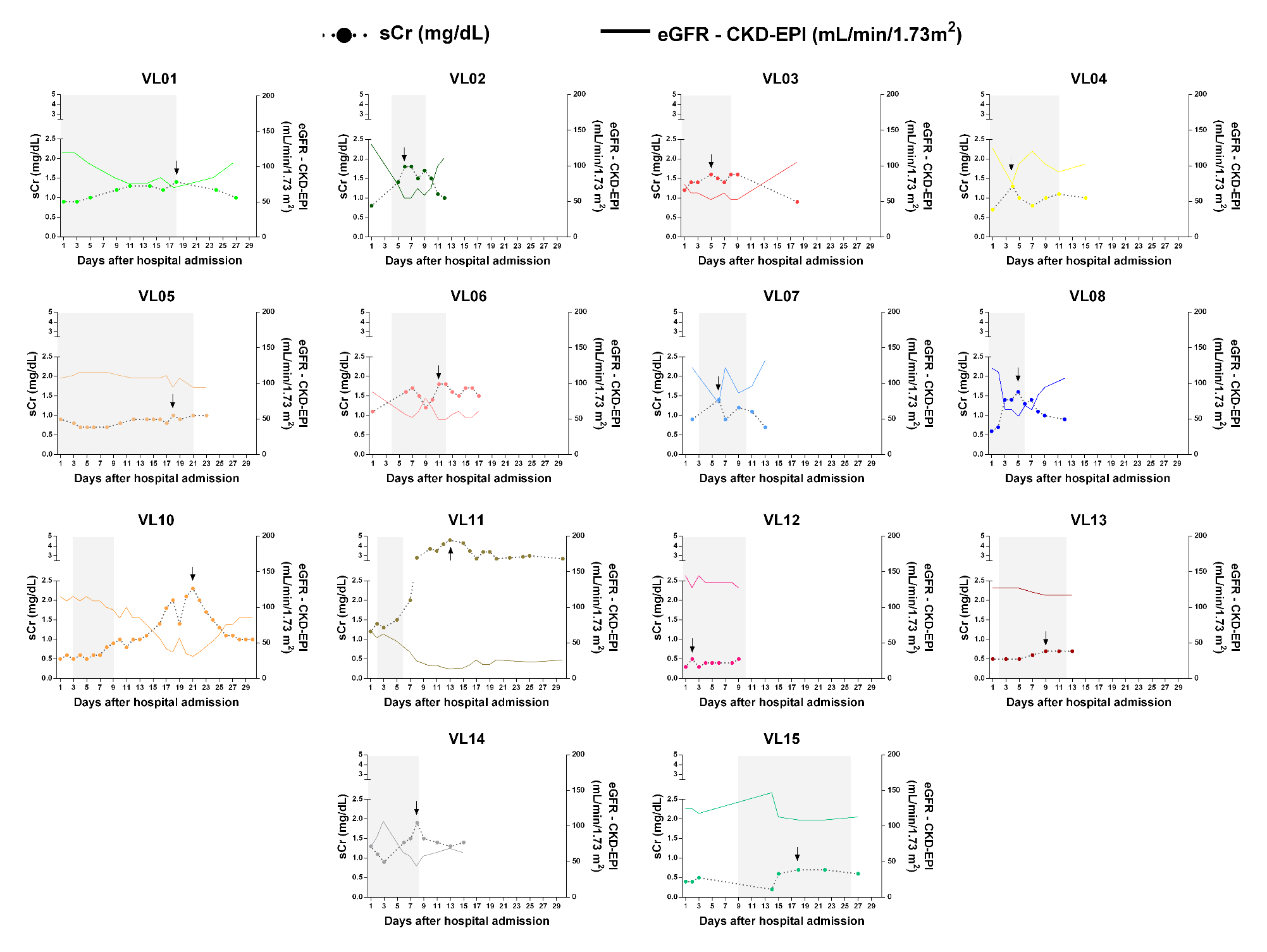


**Supplementary Figure S2: Individual temporal distribution of sCr levels and eGFR on anti-*Leishmania* treatment.** Each symbol represents sCr values recorded during the hospitalization period. The solid colored lines represent eGFR during the hospitalization period. The gray strip represents the time frame in which each patient was under VL treatment. The black arrows represent the highest sCr value recorded (sCr peak).

**
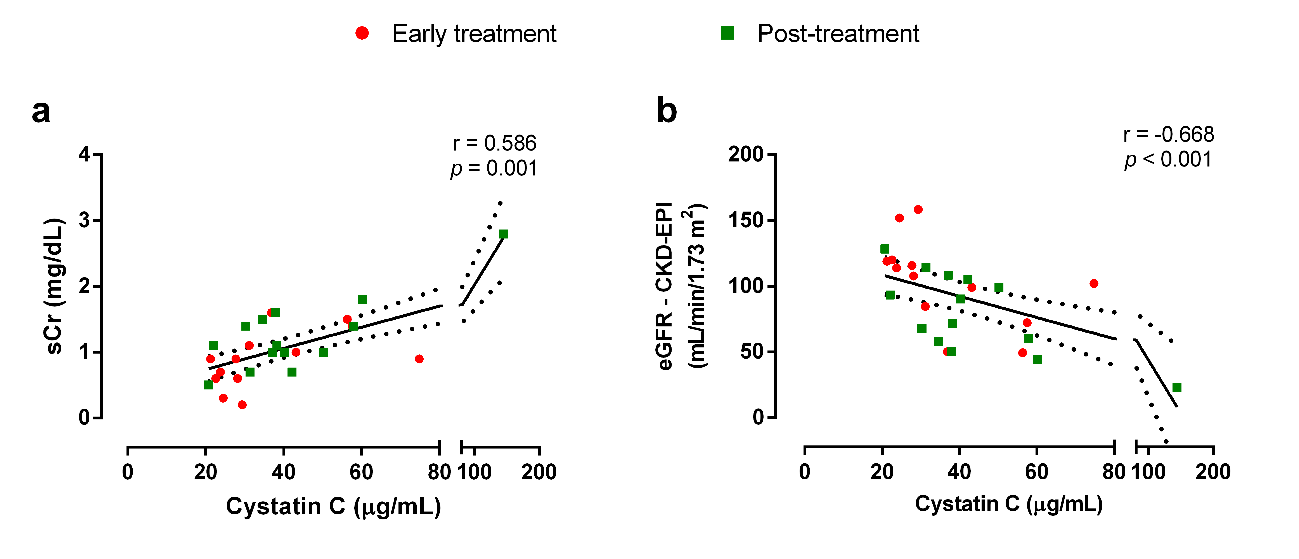
**

**Supplementary Figure S3: Correlations between renal function biomarkers in patients with VL during early and post-treatment.** Positive correlation between cystatin C and serum creatinine (A, Spearman correlation, r = 0.586, *p* = 0.001) levels. Negative correlation between cystatin C and eGFR by chronic kidney disease epidemiology collaboration equation (B, Spearman correlation, r = -0.668, *p* < 0.001).


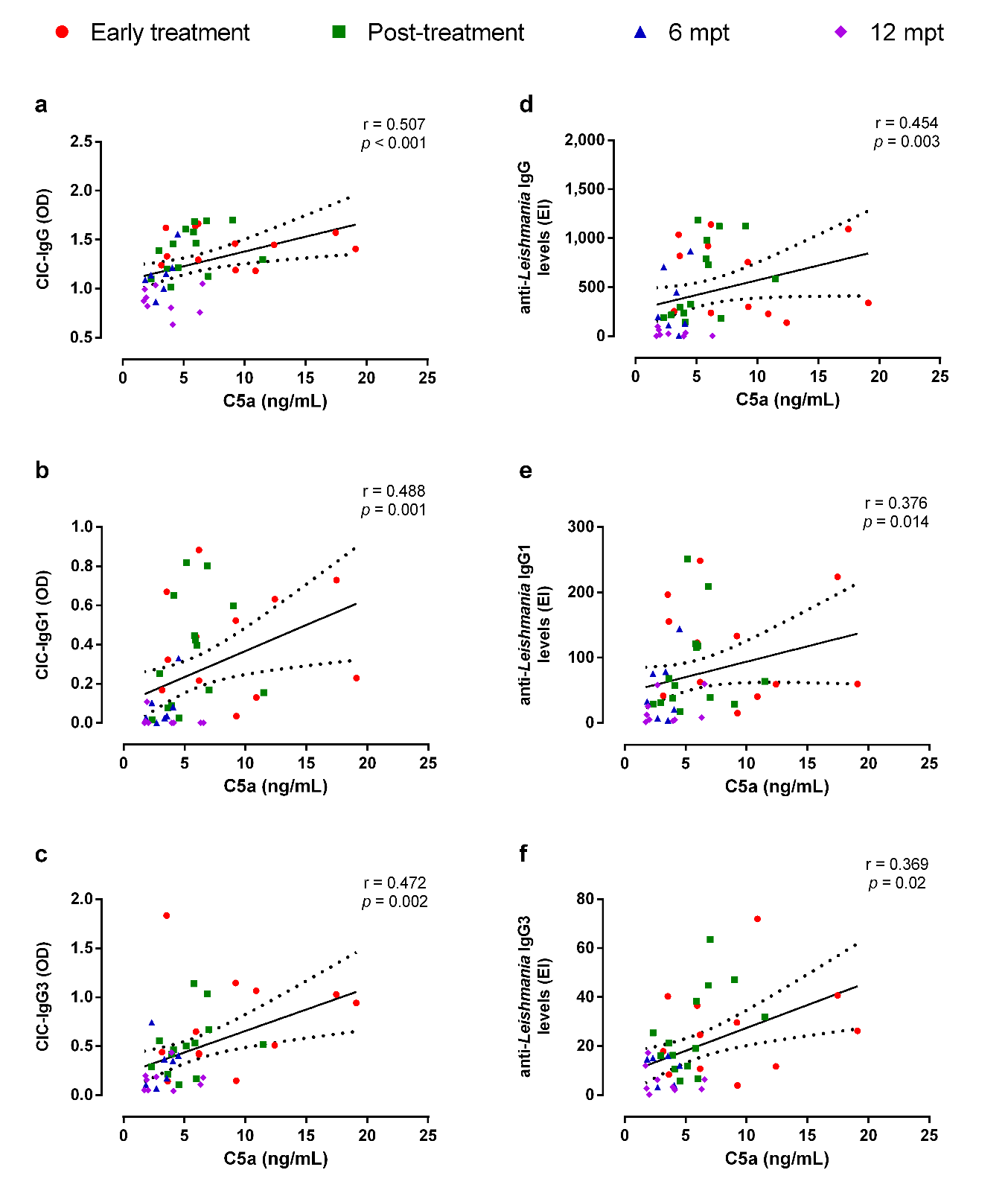


**Supplementary Figure S4: Correlation between C5a levels and circulating immune complexes or anti-*Leishmania* Igs throughout the clinical follow-up.** Positive correlation between C5a and IgG- (A, Spearman correlation, r = 0.507, *p* < 0.001), IgG1- (B, Spearman correlation, r = 0.488, *p* = 0.001) and IgG3- (C, Spearman correlation, r = 0.472, *p* = 0.002) containing immune complexes. Positive correlation between C5a and titers of anti-*Leishmania* IgG (D, Spearman correlation, r = 0.454, *p* = 0.003), IgG1 (E, Spearman correlation, r = 0.376, *p* = 0.014) and IgG3 (F, Spearman correlation, r = 0.369, *p* = 0.02).

**Supplementary Table S1:** Bivariate linear correlation analysis between cystatin C levels and inflammatory cytokines in VL patients.

|  | **Cystatin C** | |
| --- | --- | --- |
|  | ***Spearman correlation*** | |
|  | **R^2^** | ***P*** |
| 1- IL-1β | -0.165 | 0,291 |
| 2- IL-6 | 0,023 | 0,883 |
| 3- IFN- γ | 0,269 | 0,08 |
| 4- TNF | 0,264 | 0,09 |
